# RNAi Experiments in *D. melanogaster*: Solutions to the Overlooked Problem of Off-Targets Shared by Independent dsRNAs

**DOI:** 10.1371/journal.pone.0013119

**Published:** 2010-10-01

**Authors:** Erwin Seinen, Johannes G. M. Burgerhof, Ritsert C. Jansen, Ody C. M. Sibon

**Affiliations:** 1 Section of Radiation and Stress Cell Biology, Department of Cell Biology, University Medical Centre Groningen, University of Groningen, Groningen, The Netherlands; 2 Epidemiology, Faculty of Medical Sciences, University Medical Centre Groningen, University of Groningen, Groningen, The Netherlands; 3 Groningen Bioinformatics Centre, Groningen Biomolecular Sciences and Biotechnology Institute, University of Groningen, Haren, The Netherlands; Texas A&M University, United States of America

## Abstract

**Background:**

RNAi technology is widely used to downregulate specific gene products. Investigating the phenotype induced by downregulation of gene products provides essential information about the function of the specific gene of interest. When RNAi is applied in *Drosophila melanogaster* or *Caenorhabditis elegans,* often large dsRNAs are used. One of the drawbacks of RNAi technology is that unwanted gene products with sequence similarity to the gene of interest can be down regulated too. To verify the outcome of an RNAi experiment and to avoid these unwanted off-target effects, an additional non-overlapping dsRNA can be used to down-regulate the same gene. However it has never been tested whether this approach is sufficient to reduce the risk of off-targets.

**Methodology:**

We created a novel tool to analyse the occurance of off-target effects in *Drosophila* and we analyzed 99 randomly chosen genes.

**Principal Findings:**

Here we show that nearly all genes contain non-overlapping internal sequences that do show overlap in a common off-target gene.

**Conclusion:**

Based on our *in silico* findings, off-target effects should not be ignored and our presented on-line tool enables the identification of two RNA interference constructs, free of overlapping off-targets, from any gene of interest.

## Introduction

Genes can be silenced using RNA interference (RNAi). This powerful method is widely used to study biological consequences induced by the down-regulation of selected genes [1], [2], [3], [4]. Since its discovery, a great amount of valuable information has been collected using this technology. However, RNAi technology also has some drawbacks such as off-target effects [5], [6], [7], [8], [9], [10], [11], [12]. Off-target effects are caused by short stretches of sequence similarity between the RNAi molecule and one or more genes other than the target. Because of high success rates, the fly and worm (*D. melanogaster* and *C. elegans*) model systems generally use large double strand RNAs (dsRNAs) of 300–800 bp. From (large) dsRNAs, numerous siRNAs are generated by the action of DICER and each of these can provoke an RNAi response and exert their gene down-regulating action [13]. Although this results in a favourable synergistic RNAi response towards the target gene, it may in theory also increase the number of off-target possibilities.

**Figure 1:**
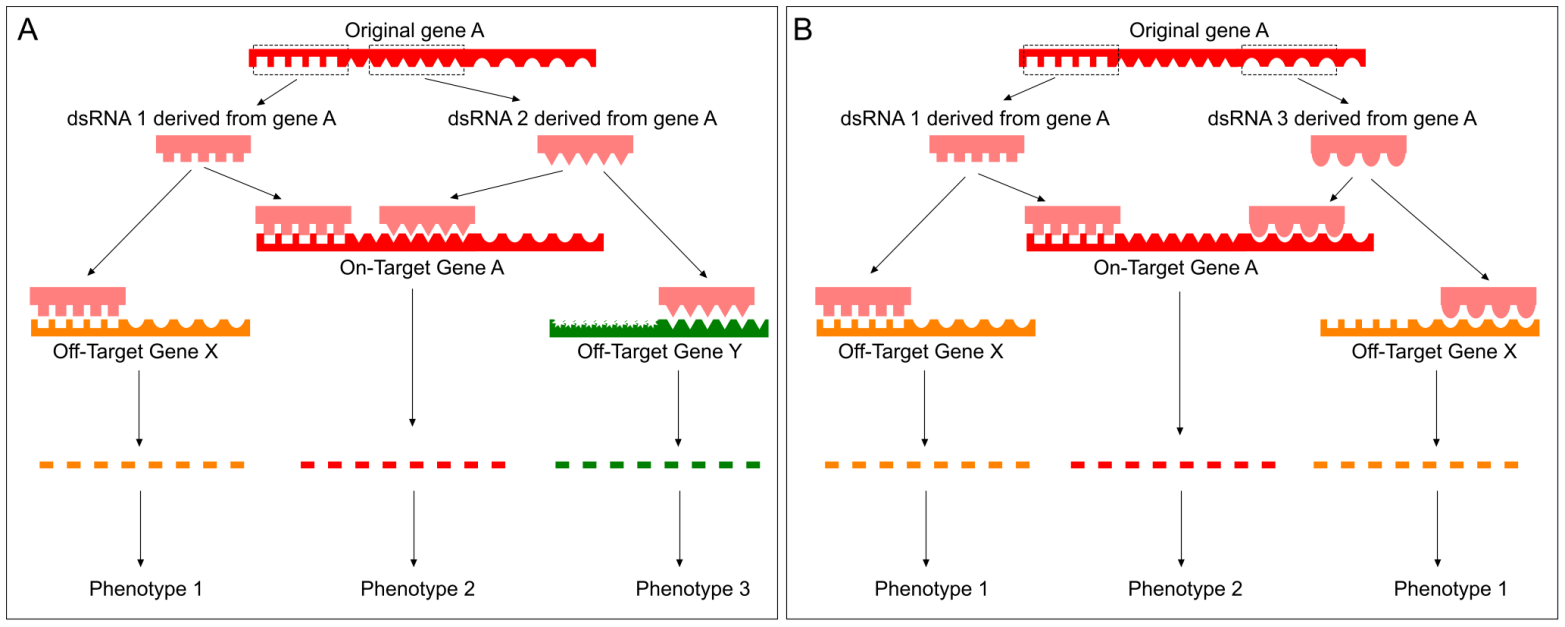
Hypothetical off-target events. **A**: Schematic presentation of the event in which identical phenotypes are induced because of shared on-target effects and at the same time different phenotypes are induced because of off-target effects. Phenotype 2 is due to down-regulation of the on-target gene and is induced by dsRNA1 and dsRNA2. Phenotype 1 and 3 are due to down regulation of the off-target gene X and Y respectively and are specific for the individual distinct dsRNAs. In this fortunate event, the individual off-target effects are not identical and are classified as off-target-effects; *bona fide* conclusions will be drawn from the outcome of this experiment. **B**: Schematic presentation of the event in which identical phenotypes are induced because of shared on-target effects but at the same time an additional identical phenotype is induced by the use of the two independent dsRNAs caused by off-target effects. Phenotype 2 is due to down-regulating the on-target gene and is shared by dsRNA1 and dsRNA3. Phenotype 1 is due to down regulation of a shared off-target gene of the distinct dsRNAs. In this unfortunate event, the off-target effects are identical and will be classified as on-target effects; false conclusions will be drawn from the outcome of this experiment.

A straightforward method to reduce off-target effects, is to use 2 independent and non-overlapping dsRNAs to down-regulate a specific target. Because these dsRNAs are different in sequence composition, their individual off-targets are also assumed to be unique while they both silence the same on-target gene. Consequently, it is reasonable to assume that any shared phenotype which is observed after the independent use of both dsRNAs is an effect of down-regulating the on-target gene (ure 1A). Although, this line of reasoning is rational, hypothetically it is possible that different non-overlapping siRNAs may actually target different sequences within one identical off-target gene (illustrated in Figure 1B). In such an unfortunate case, a shared off-target effect induced by 2 independent dsRNAs may be misinterpreted as an on-target effect. It has never been investigated what the occurrences are of shared off-target effects when dsRNA are randomly chosen. Here, we present a detailed analysis, based on sequence similarity and a randomized trial which suggest that most genes have independent dsRNA-spanning sequences showing sequence similarity with the same off-target gene. In addition, we present an on-line tool that allows to scan *Drosophila* gene sequences for the occurrence of off-target overlapping regions and to design dsRNAs that have a reduced likelihood to induce identical off-target effects.

## Results and Discussion

**Figure 2:**
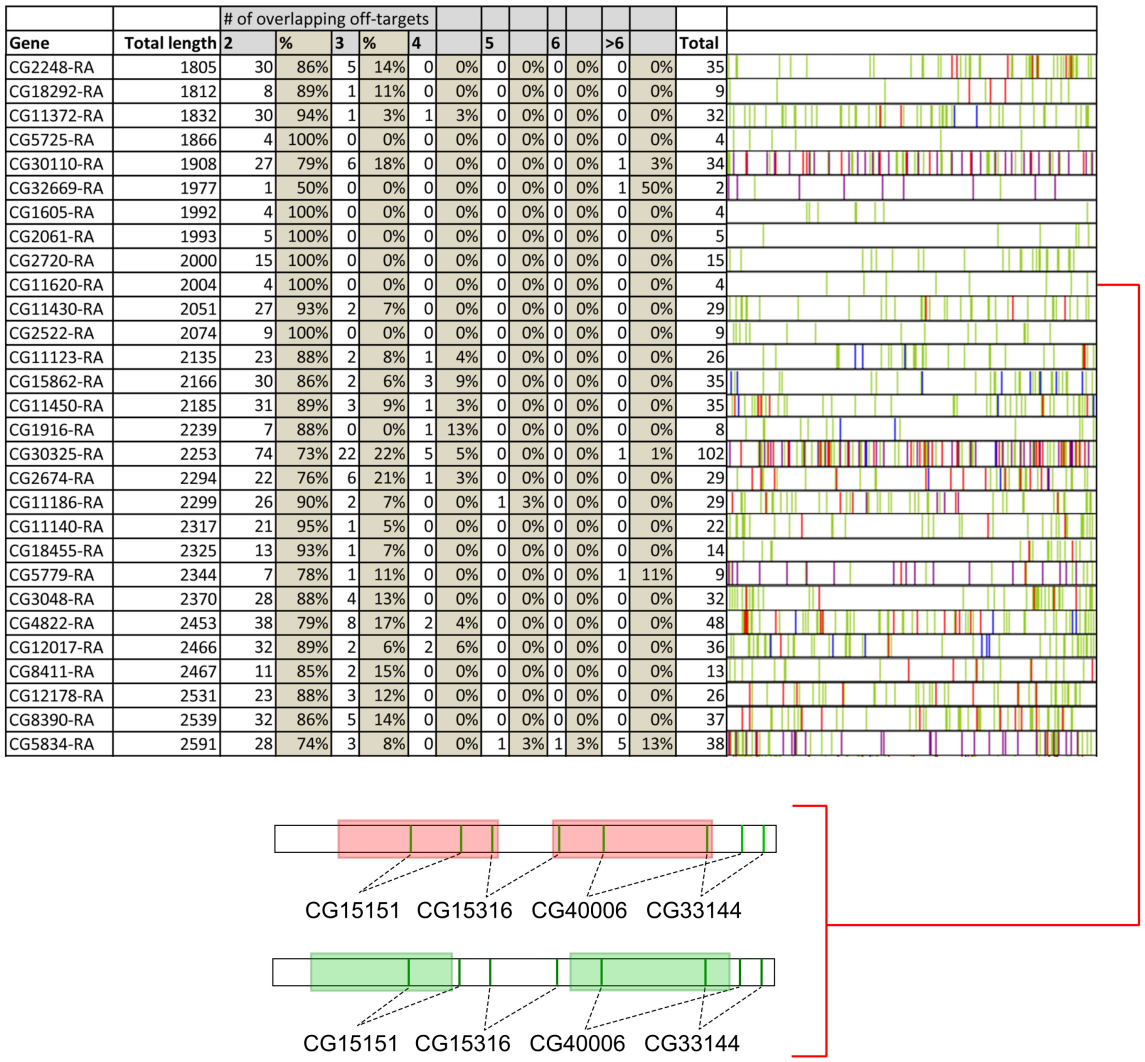
Non-overlapping cDNA sequences of randomly chosen genes have a high prevalence of sharing off-targets in pre-mature sequences of other genes. Example of genes for which –based on sequence similarities- overlapping off-targets exist. The number of items per off-target group is given both in numbers and percentages. As an example: the cDNA of gene CG11372-RA contains 30 events of sequences with a shared off-target and 1 event of triplicate sequences that share the same off-target and 1 event of quadruple sequences that share the same off-target. Green lines represent sites that share an identical off-target with one other site, red lines represent sites that share an identical off-target with 2 other sites, and blue lines with 3 other sites. Purple lines represent sites that share identical off-targets with 5 or more other sites. The complete report from the 99 randomly selected genes is presented in Supporting Information S1. Note that for some genes the lines representing the off-target events are in close proximity and cannot be distinguished as separate lines in this illustrative figure. Overall, there appears to be a tendency for the occurrence of overlapping off-targets at the boundary (UTR's) of the genes, as is evident in for example CG5834 (last gene in the list). The insert shows a more detailed illustration for the analysis of the cDNA of gene CG11620. The green vertical lines represent sites that share an identical off-target with one other site. Sites that share the same off-target are connected with dotted lines and the shared off-target (as CG number) is indicated for each pair. To avoid off-target effects, dsRNA constructs should be chosen in such a way that the dsRNA constructs do not include both members of one pair. The green boxes represent areas which do not include both members of one pair. In order to reduce the likelihood of shared-off target effects, dsRNAs should be designed using sequences from the green regions. In contrast the red areas do include both members of one pair. When 2 independent dsRNA constructs will be designed from these areas, these dsRNA constructs do share sequence similarities with the same off-target gene. Our tool provides for all the genes present in the Drosophila genome the green areas.

**Figure 3:**
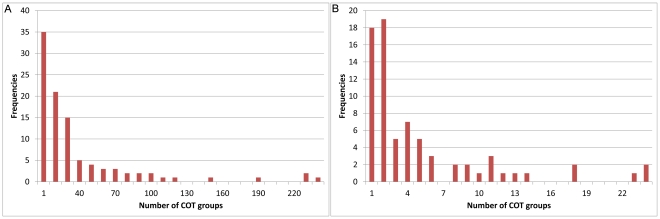
Common Off-Target (COT) groups are present in all 99 analized genes. Cot-group frequency distribution is given for all 99 analyzed genes. A cot-group consists of 2 or more siRNAs, derived from a single gene, that map to the same off-target gene. The x-axis shows the number of cot-groups, the y-axis shows the frequency of genes that contain that number of cot-groups. **A**: On average, 42 cot-groups were found per gene with a large spread (SD = 48.579; N = 99) because of the high correlation with gene length (see figure 4). **B**: Filtering for introns shows that the COT group frequencies are much lower, mostly concentrating around 1–3. Figure 6 and Supporting Information S1 illustrate the cot-groups per gene after filtering out intron targets.

**Figure 4:**
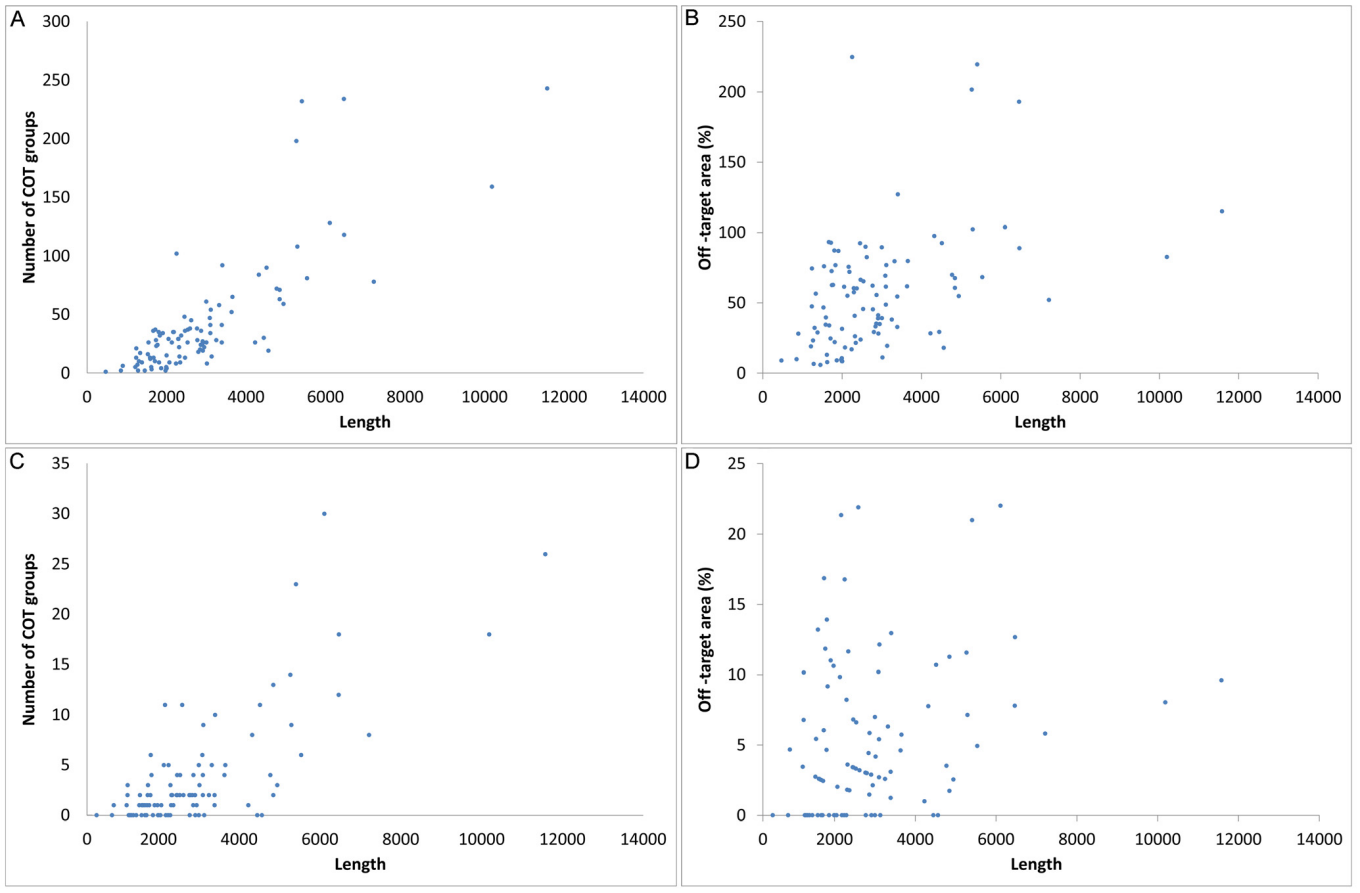
The number of cot-groups correlate with the length of the cDNA. As expected, the length of the gene correlates with the number of cot-groups that can be formed (Spearman's correlation coefficient of 0.44; P < 0.001). This is because the number of potential siRNAs that may originate from one cDNA sequence increases proportionately with the length of the gene. Each potential siRNA adds up on the possibility to form a cot-group. **A**: The number of cot-groups against the length of the gene is plotted. **B**: The percentage of the sequence within the analyzed genes that map to common off-targets is shown. These values were acquired by multiplying the members of the different cot-groups with the length of a single siRNA (21-bp) within every gene and this is divided by the whole gene length. These values become less reliable with large genes, because there is an increased chance of a single siRNA being part of multiple cot-groups. In that case the area may reach beyond the 100%. **C-D**: An identical analysis as described under A and B was performed but now only off-targets were considered targeting mature RNA sequences .

Statistical analysis on a randomized genome shows that it is likely that 2 distinct 21 nt sequences from the same gene can map closely elsewhere on the genome (see Supporting Information S1). This hypothetical event (illustrated in Figure 1B) may cause distinct dsRNAs to have common off-targets and that particular combinations of dsRNAs should therefore be avoided. These calculations are based on a non-organized genome containing random sequences, while the *Drosophila* genome is highly functional and far from ‘randomized’. To evaluate the risks of our hypothetical event more pragmatically, we used the following approach. First, we picked a dataset of 99 random chosen genes (see Supporting Information S1) from the *D. melanogaster* genome. We investigated the occurrence of independent dsRNAs derived from one gene to have shared off-targets. dsRNAs are often derived from cDNA so for our analysis only the cDNA of the 99 genes were considered. Because the complete cDNA can be used to design dsRNAs from, and the dsRNAs are split into siRNAs of approximately 21nt by the RNAi machinery, we first created a list of all possible siRNA sequences that can be obtained from the cDNA sequences of each of these 99 genes. This complete list was subsequently reduced using established scoring rules to exclude 21-bp siRNAs that are most likely non-active (see Supporting Information S1). We like to stress that this assumption will only underestimate our findings. Next we calculated the occurrence of all siRNA derived from one cDNA to have a shared off-target with another siRNA derived from the same gene. For this analysis we included pre-mRNA sequences of the complete *D. melanogaster* genome because of the following published results: 1) It has been demonstrated that the RNAi machinery can target pre-mRNAs in *C. elegans* [14]. 2) RNA silencing components have been shown to localize in the nucleus in other organisms (including human) [15], [16], [17], [18], [19], [20], [21], [22], further suggesting that pre-mRNAs can be targetted by the RNAi machinery. 3) RNAi constructs can be complementary to miRNAs which are often derived from introns [23] and might act like antagomirs [24]. We therefore analysed the filtered list of siRNA sequences against both mature and pre-mature RNA sequences to map all possible off-targets with up to 3 mismatches in their sequence alignments with the use of a new tool (see Methods and http://www.RNAiSelect.info/dsrna). By doing so, a list of potential off-targets for each of the individual genes was generated. Next we analyzed whether there was overlap between the potential off-targets of siRNAs derived from the same gene (see Methods). We used the term *cot-group* (Common Off-Target group); a cot-group consists of 2 or more siRNAs, derived from a single gene, that map to the same off-target gene (also illustrated in Figure 2; the lines represent members of cot-groups) . The generated siRNA lists of all 99 genes were scanned individually for the presence of cot-groups. The occurrence of cot-groups appeared to be present in all genes, with sometimes excessive high frequencies (Figure 3). As expected, the number of cot-groups are highly correlated with the length of the cDNA of the gene; the larger the sequence, the more cot-groups are formed (Figure 4).

**Figure 5:**
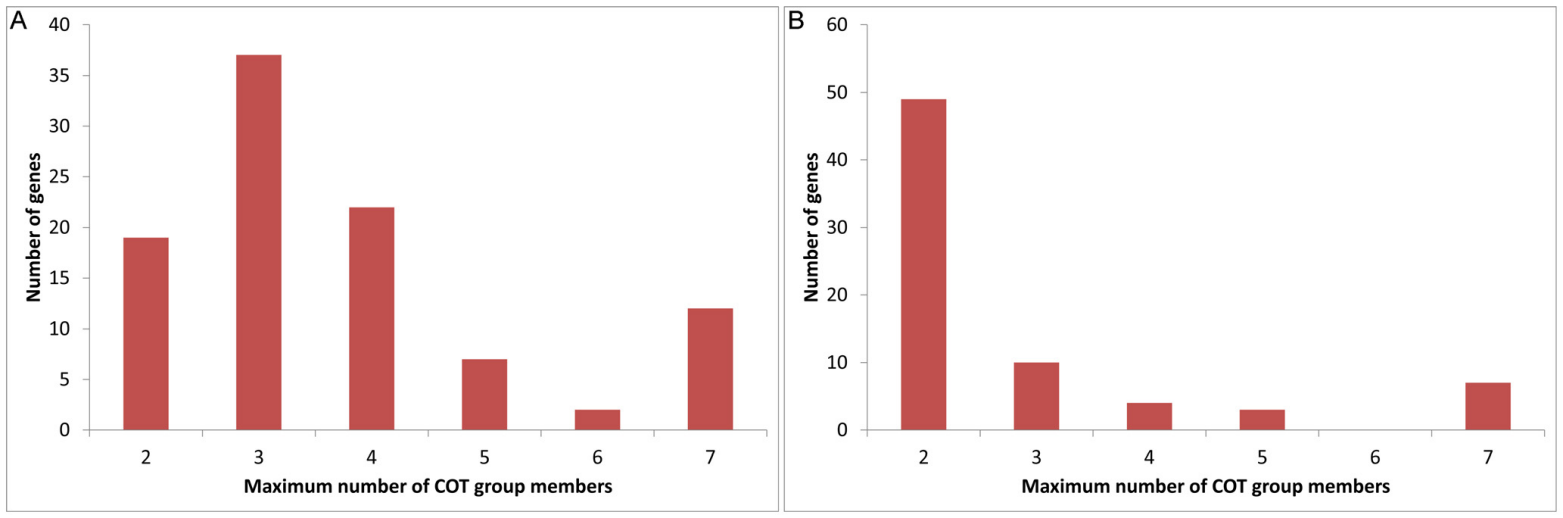
Most genes have cot-groups with at least 2 members. **A**: Distribution of the maximum cot-group size for the 99 analyzed genes. For each cot-group with the indicated number of members on the x-axis, the number of genes where counted that have cot-groups with a corresponding maximum cot-group size. This shows for example that there are 19 genes that have cot-groups with no more than 2 members. Most genes (38) in our analysis appear to have at least one cot-group present with up to 3 members. Due to the correlation between gene length and number of cot-groups, some large genes in our analysis also show very large cot-groups which account for the unexpected large 7+ count as plotted in the last bar (see also Figure 2 and Supporting Information S1). **B**: The results after filtering out intron targets are presented, showing that most genes have at least one cot-group with 2 members.

We then looked at the general profile of the cot-groups for each gene separately and tried to deduce the required number of dsRNAs to strongly reduce the event of common off-targets. If for example the number of members within the cot-groups does not exceed 2, than this implies that at most 2 siRNAs within the same gene map to a common off-target. For that particular situation, the use of 3 or more non-overlapping dsRNA will always generate bona fide data as there is no possibility for all 3 of them to share a common off-target (bases on sequence similarity). Unfortunately, most genes have cot-groups with at least 3 members (Figure 5, also depicted by red lines in Figure 2 and in Supporting Information S1) or even 4 members (Figure 5, also depicted by blue lines in Figure 2 and in Supporting Information S1). This finding demonstrates that just using multiple non-overlapping dsRNAs is not sufficient to exclude off-target events (see also insert Figure 2), even if the number of independent dsRNAs is 3 or more. We therefore developed a bioinformatics approach to design dsRNAs that do not contain predicted off-targets. Our freely available website presents such a tool at http://www.RNAiSelect.info/dsrna. This web based tool accepts a gene name as input and presents a number of choices each containing a combination of 2 unique dsRNAs that lack overlapping –based on sequence similarity- off-targets (Supporting Information S1).

**Figure 6:**
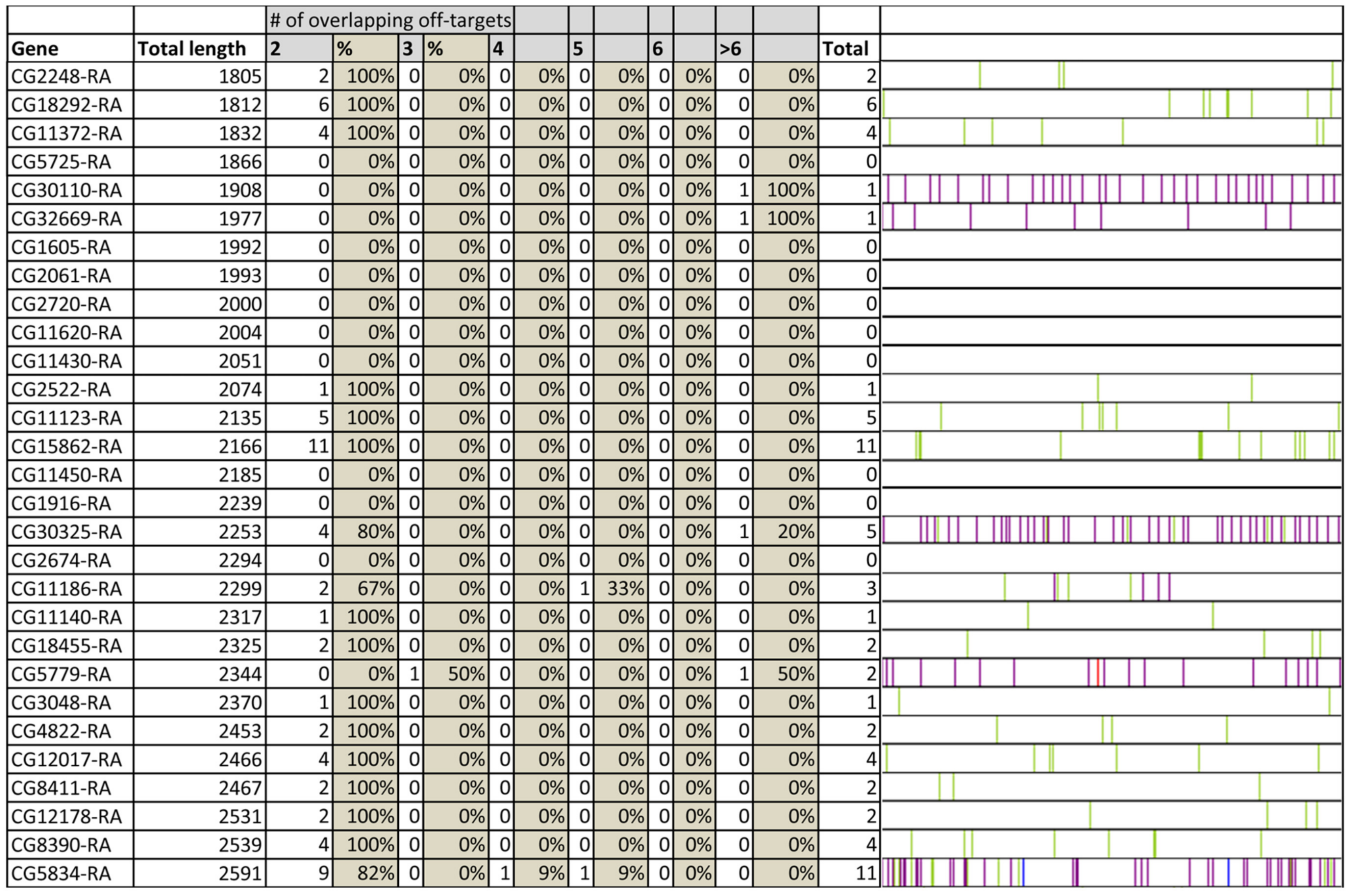
Non-overlapping cDNA sequences of randomly chosen genes have a significant prevalence of sharing off-targets in exon derived sequences of other genes. All 99 cDNA sequences were re-analyzed, now ignoring any predicted off-targets that occur within intron sequences. Although this lowers the predicted off-targets, it still shows a significant occurrence of overlapping off-targets. Our analysis shows that after applying this filter, 74% of the genes contain non-overlapping cDNA sequences that share sequence similarity with the same off-target (see also Supporting Information S1).

Next, we repeated the above analysis, but now only considering off-targets targeting mature RNA sequences, because these are maybe be more active in RNAi [25]. Filtering out the intron off-targets, causes much less off-targets to be found in general per cDNA (Figure 6). Overall, both the sizes and occurrences of the COT groups are smaller (Figure 3B, Figure 5B). Nevertheless, there is still a significant number of overlapping potential off-targets to be expected in >74% of the genes. In 24% of the analysed genes there is at least 1 COT groups present of size 3 (Figure 5B), meaning that there are at least 3 areas within the cDNA that target the same off-target. Therefore, even if only mature RNA sequences are considered and 2 randomly chosen non-overlapping dsRNA's are used, the experimental outcome can be obscured by off-target effects. This further underscores the utility of our tool.

Although not exclusively, we observed a strong tendency for overlapping off-targets to occur at the end of genes (see graphical illustrations of the common off-targets in Figure 2 and in Supporting Information S1), corresponding to the untranslated regions (UTR). The UTR sequences are less unique in the genome as compared to the coding region and therefore preferably should be avoided when dsRNA constructs are designed. Our tool includes an option to avoid UTR sequences to minimize off-targets when designing dsRNAs of interest.

Our analysis demonstrated that most genes in the *D. melanogaster* genome contain 2 or more (distinct) sequences that show sequence similarity (containing 3 or less mismatches) to the same off-target gene. The potential consequence of these overlapping occurrences is that 2 dsRNAs which are generated to down-regulate a specific target gene, may possess a common off-target gene as well. In case these 2 distinct dsRNAs are used, their common phenotype induced by down-regulation of their shared off-target gene may lead to misinterpretation of the experiment. We present a method to identify 2 distinct dsRNAs from a gene of choice that do not show any off-target overlap, -based on sequence similarity- by performing a thorough off-target overlap analysis. This tool is freely available at http://www.rnaiselect.info/dsrna and can be used by the *Drosophila* community where dsRNAs are generally used for gene down-regulation.

## Methods

Genomic data (build 45–43b for *Drosophila*) were downloaded from the Ensembl website (www.ensembl.org). The data from Ensemble and its derived seed tables were processed, stored and indexed in a MySQL database, version 5.0, running on top of Ubuntu 6.06. The on-line available RNAiSelect program (http://www.RNAiSelect.info/) was written in C#.NET and performs a comprehensive sequence alignment against the input genome for up to 3 mismatches.

The RNAiSelect algorithm was specifically designed for finding relationships between short nucleotide sequences. It has a high performance and usability for short-sequence studies, including siRNA (off-)targets. The complete source code and documentation for a standalone version of this algorithm may be downloaded from http://rnaiselect.sourceforge.net/. The algorithm is based on the following assumption:

“An example sequence TTTTAATTTGGGCCCGGG consists of 18 nucleotides and may be split into two 9-nt child sequences; TTTTAATTT and GGGCCCGGG. By plain observation, we know that the sequence GGGCCCGGG is exactly 9-nt separated from TTTTAATTT in the original sequence.”

For the RNAiSelect algorithm to work, we first wrote a program that generates a seed table which holds the exact genomic location(s) for every possible 9-nt sequence (4∧9, or 262.144 sequences). Generating such an index is a general strategy used by many algorithms to rapidly look-up any sequence of fixed length for its positions in the genome. The used algorithm however uses a novel method to calculate the positional relationship between indexed seeds, instead of performing string-to-string comparisons for every nucleotide after a hit has been found. In other words, by searching 9-nt subsequences of the whole query sequence for consecutive matches of locations, it will find hits larger than 9 nt without performing actual DNA comparisons. This following example, in layman code, shows how to find an 18-nt sequence in the genome by first splitting the sequence into its two 9-nt subsequences and comparing these sequences with the available index table with a word size of 9.

- 1 SPLIT QUERY SEQUENCE(18 nt) INTO *dnacode_left(9 nt)* AND *dnacode_right(9 nt)*
- 2 EXTRACT LOCATIONS FROM *index_table* FOR dnacode_left AND STORE IN *seedtable_left*
- 3 EXTRACT LOCATIONS FROM *index_table* FOR dnacode_ *right* AND STORE IN *seedtable_* right
- 4 SELECT ALL HITS WHERE (LOCATIONS *seedtable_left + 9) EQUALS (LOCATIONS seedtable_right)*

This example merely demonstrates how to find an exact 18-nt hit not allowing any mismatches. However, mismatches may be added by expanding the seed searches with variations so that all possible combinations will be found. We thus included variations of the 9-nt sub-sequences and then compared the distance relationship between the original locations of the seed hits, which has to be exactly 9. This may considerably increase the number of seed searches, but because these are relatively cheap in terms of processing time, the overall performance is very high while it guarantees that every possible alignment is evaluated.

The cDNA sequences (meaning only sequences derived from the mature RNA) from each of the 99 genes (Supporting Information S1) were first analyzed for potentially active sequences as might be produced by endogenous DICER. A scoring schema was used (Supporting Information S1) during this analysis to estimate and extract the most potential sequences. Each extracted sequence was analyzed for potential off-targets. The combined output from all these potential off-targets was cross-referenced with each other to map areas on the original cDNA sequence that are predicted to have overlapping off-targets. At the same time, regions can be identified that lack these areas and dsRNA sequences can be extracted that are completely devoid of off-targets. The results are presented and 2 or more dsRNA are indicated that originate from the same gene and that are predicted to lack overlapping off-targets. From the indicated areas, dsRNA can be designed. An identical analysis can be done for every Drosophila gene of interest through a web-interface at http://www.rnaiselect.info/dsrna which presents the output in a user friendly interface.

## Supporting Information

Supporting Information S1 Statistical analysis on a randomized genome shows that it is likely that distinct sequences from the same gene map closely elswhere on the genome, demonstrating that non-overlapping dsRNA constructs derived from the same gene can share a common off-target. 99 randomly-chosen genes were analysed for these -based on sequence similarity- off-target effects using defined scoring rules. Our results show that non-overlapping cDNA sequences of randomly chosen genes have a high prevalance of sharing off-targets in pre-mature and in exon sequences of other genes. A freely available website is provided to avoid these off-target effects and to identify “clean” combinations of dsRNA constructs derived from the same gene to down regulate any gene of interest.
(6.68 MB DOC)
