# Supplementary material for: RNAi Experiments in D. melanogaster: Solutions to the Overlooked Problem of Off-Targets Shared by Independent dsRNAs
Source: PLoS One. 2010 Oct 1;5(10):e13119. doi: 10.1371/journal.pone.0013119 (PMC2948504; doi:10.1371/journal.pone.0013119)
Supplement: Supporting Information S1 — Statistical analysis on a randomized genome shows that it is likely that distinct sequences from the same gene map closely elswhere on the genome, demonstrating that non-overlapping dsRNA constructs derived from the same gene can share a common off-target. 99 randomly-chosen genes were analysed for these -based on sequence similarity- off-target effects using defined scoring rules. Our results show that non-overlapping cDNA sequences of randomly chosen genes have a high prevalance of sharing off-targets in pre-mature and in exon sequences of other genes. A freely available website is provided to avoid these off-target effects and to identify “clean” combinations of dsRNA constructs derived from the same gene to down regulate any gene of interest. (6.68 MB DOC) [file pone.0013119.s001.doc]

**Supplementary file**

To investigate the probability of shared off-targets, we first addressed the question whether a random sequence will likely match or nearly match to any other sequence present in the *D. melanogaster* genome. To estimate this, a formula previously designed by others for the same purpose [14], was used. With this formula we calculated what the occurrence is of any random sequence of 21 nt to find one other (k=2) identical sequence within the genome. For this calculation, the 21 nt sequence and the complete genome are for the sake of simplicity considered to consist of random sequences. In this formula, *n* is the number of possible different sequences of a specific sequence length, equal to 4^21^ for a sequence of 21 nt long (assuming 4 possible different nucleotides; A, T, C or G). 21 nt was chosen because siRNAs of this size may be generated when large dsRNAs are used in RNAi experiments for the *D. melanogaster* genome. The number of copies to be present is represented by k (=2).

(1)
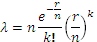


The size of the Drosophila genome is 168.7 Mb (r), therefore it can be expected that, within a randomized genome of this size, there are 3,235 occurrences (*λ*) of random 21 nt sequence that exactly match to another sequence within that same genome. However, in this estimation mismatches are neglected while sequences containing mismatches may also contribute to a measurable RNAi response [15-18]. When mismatches are taken into account, this considerably increases the number of different possibilities for an siRNA to find successful sequence hybridization partners with other mRNAs. The number of sequences that are possible with length *n* and up to *k* mismatches is calculated with the standard formula:

(2)
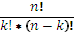


Note that in this standard formula the symbols *n* and *k* are also used, however they are different from *n* and *k* presented in formula 1. We used 3 mismatches in this and following calculations as this has been known to elicit a successful RNAi response [2]. Using *k=3* results in 1,330 different combinations for a 21-bp sequence. Combining this with formula (1), *n* is divided by 1,330 and *λ* is recalculated to be 4,089,178 occurrences instead of the previous calculated 3,235 for exact matches. Each occurrence may span up to 42-nt (2x the size of a single occurring siRNA; 21-nt), and therefore all events together (42 x 4,089,178 ) may cover the complete genome.

# Supplementary Figure 1

**A freely available website to identify “clean” dsRNA partners**

Illustration of the on-line website (www.rnaiselect.info/dsrna), enabling the identification of dsRNA constructs that do not have shared off-targets. The table lists different dsRNAs combinations (red and blue boxes) that do not have predicted overlapping off-targets (off-targets are indicated by vertical lines) on top of a schematic view of the complete cDNA sequence. By using these dsRNA sequences, the chances that the experimental outcome is influenced by shared off-target effects will most likely be reduced.

# Supplementary Table 1

| Description | Score |
| --- | --- |
| 30%-52% GC Content | 1 point |
| 3 or more A/Us at positions 15-19 | 1 point per AU |
| Tm>20°C | 1 point |
| A at position 19 | 1 point |
| A at position 3 | 1 point |
| U at position 10 | 1 point |
| G/C at position 19 | -1 point |
| G at position 13 | -1 point |
| >4 sequential nucleotide repeat | -9 points |
| > 4 diplet repeat | -9 points |

**Description of scoring rules**

Scoring scheme used to define most potent siRNAs, based on a summary from several publications. Only sequences scoring at least 6 points were considered.

References:

- http://www.protocol-online.org/prot/Protocols/Rules-of-siRNA-design-for-RNA-interference--RNAi--3210.html

- Jagla, B., Aulner, N., Kelly, P.D., Song, D., Volchuk, A., Zatorski, A., Shum, D., Mayer, T., De Angelis, D.A., Ouerfelli, O. et al. (2005) Sequence characteristics of functional siRNAs. RNA, 11, 864-872.

- Reynolds, A., Leake, D., Boese, Q., Scaringe, S., Marshall, W. and Khvorova, A. (2004) Rational siRNA design for RNA interference. Nature biotechnology., 22, 326-330.

- Elbashir, S.M., Martinez, J., Patkaniowska, A., Lendeckel, W. and Tuschl, T. (2001) Functional anatomy of siRNAs for mediating efficient RNAi in Drosophila melanogaster embryo lysate. EMBO J, 20, 6877-6888.

**Supplementary Table 2**


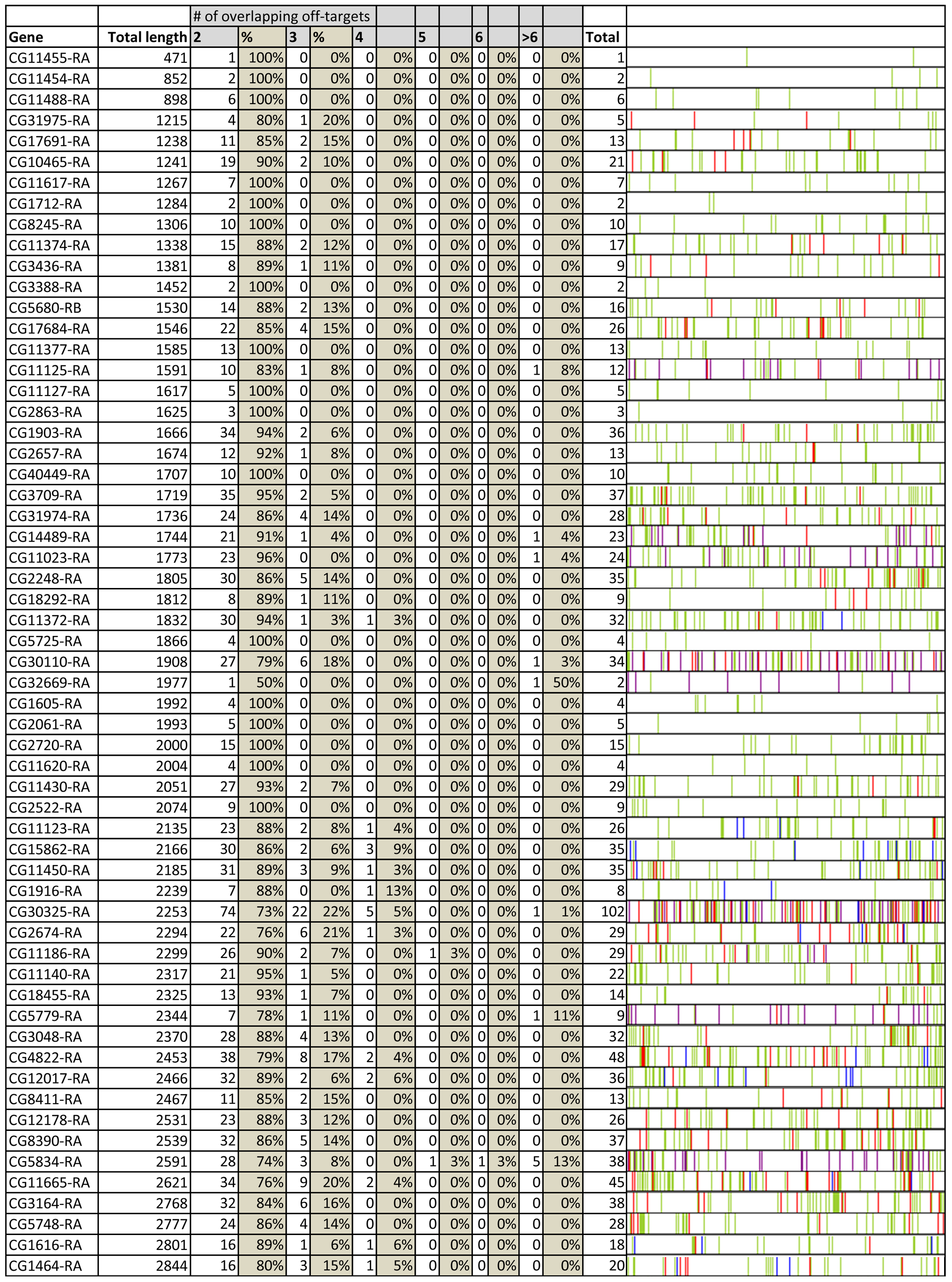


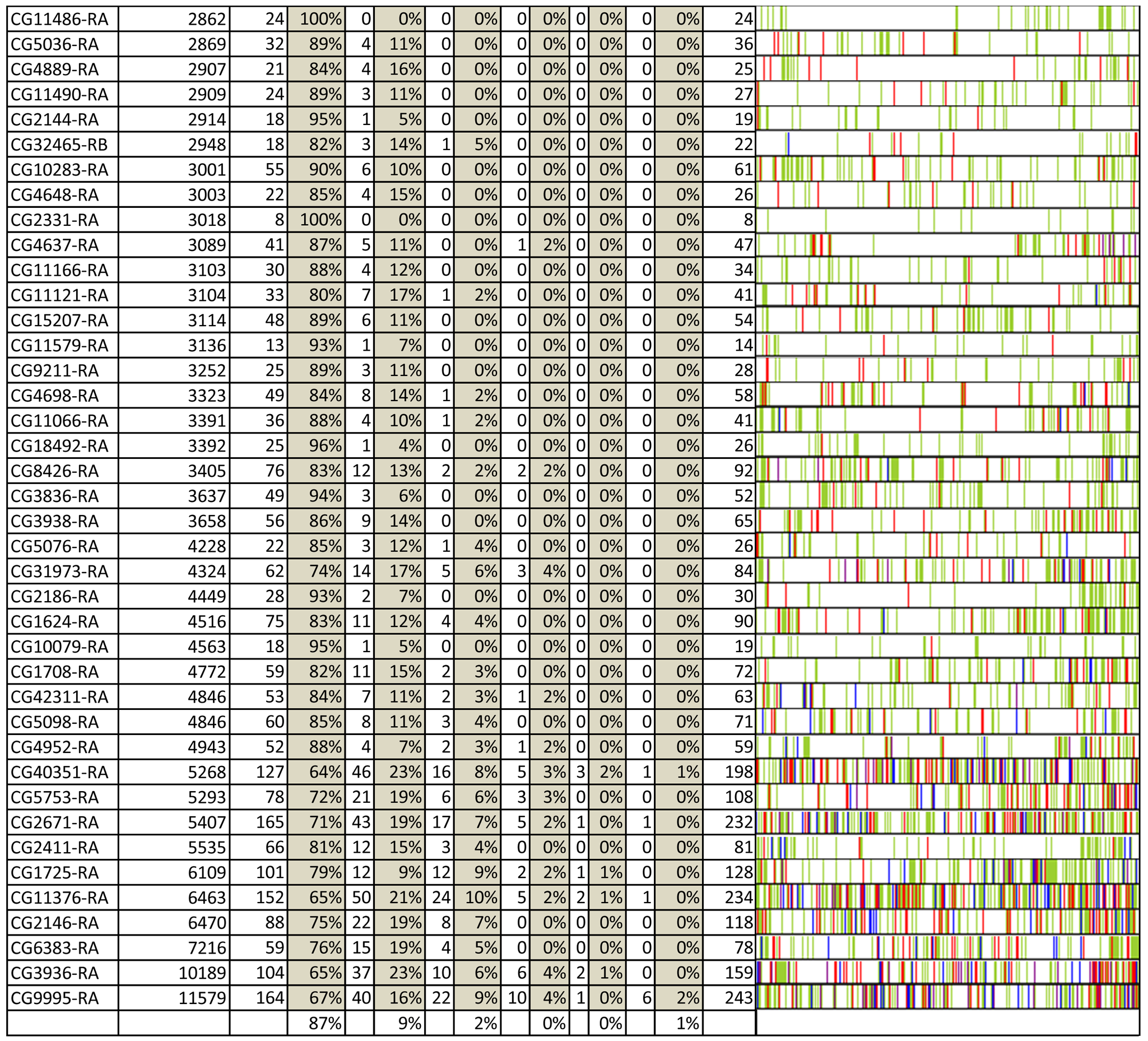


**Non-overlapping cDNA sequences of randomly chosen genes have a high prevalence of sharing off-targets in pre-mature sequences of other genes**

Detailed report of all 99 analyzed genes showing the overlap between sequence similarities based off-targets. The number of items per off-target group is given both in numbers and percentages. As an example: the cDNA of gene CG11372-RA contains 30 events of duplicate sequences with a shared off-target and 1 event of triplicate sequences that share the same off-target and 1 event of quadruple sequences that share the same of target. Green lines represent sites that share an identical off-target with one other site, red lines represent sites that share an identical off-target with 2 other sites, and blue lines with 3 other sites. Purple lines represent sites that share identical off-targets with 5 or more other sites. Note that for some genes the lines representing the off-target events are in close proximity and cannot be distinguished as separate lines in this illustrative figure.

# Supplementary Table 3


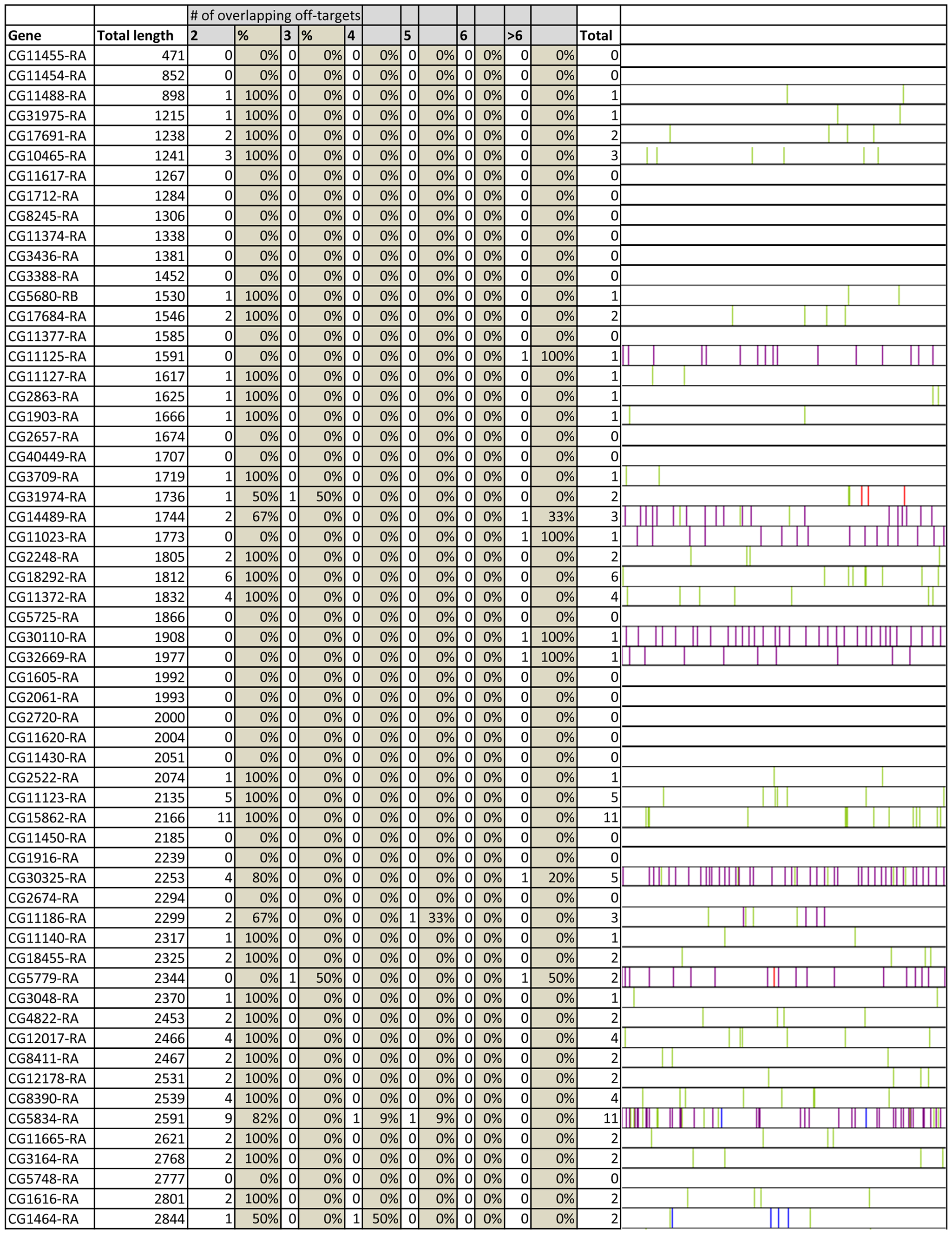


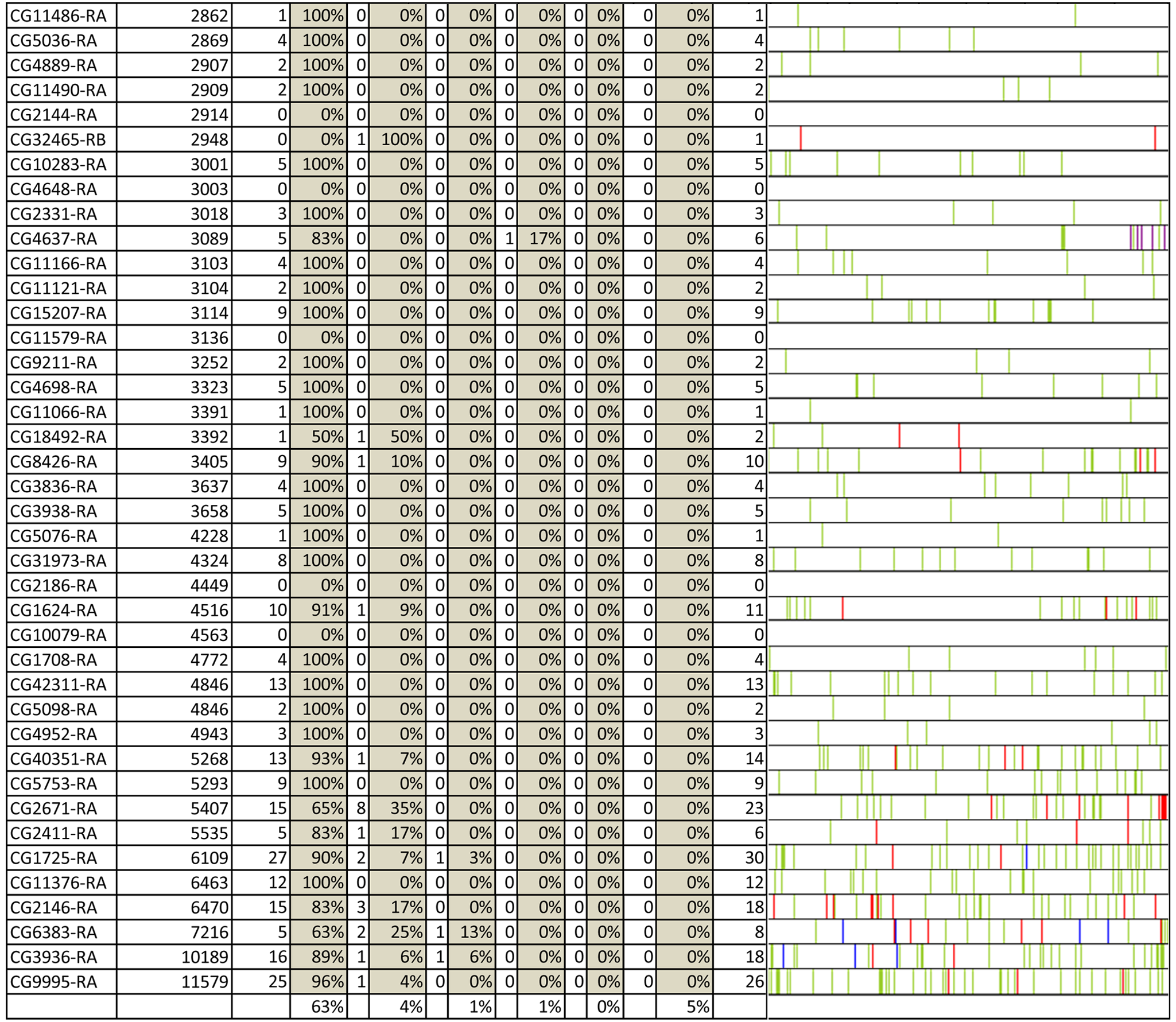


**Non-overlapping cDNA sequences of randomly chosen genes have a significant prevalence of sharing off-targets in exon derived sequences of other genes**

All 99 cDNA sequences were re-analyzed, now ignoring any predicted off-targets that occur within intron sequences. Although this lowers the predicted off-targets, it still shows a significant occurrence of overlapping off-targets. Our analysis shows that after applying this filter, 74% of the genes show off-targets that occur more than once within the same derived cDNA sequence. The number of items per off-target group is given both in numbers and percentages. Green lines represent sites that share an identical off-target with one other site, red lines represent sites that share an identical off-target with 2 other sites, and blue lines with 3 other sites. Purple lines represent sites that share identical off-targets with 5 or more other sites. Note that for some genes the lines representing the off-target events are in close proximity and cannot be distinguished as separate lines in this illustrative figure.
